# Postanesthetic Cold Sensibility Test as an Indicator for the Efficacy of Inferior Alveolar Nerve Block in Patients with Symptomatic Irreversible Pulpitis of Mandibular Molars

**DOI:** 10.1155/2021/9913221

**Published:** 2021-06-21

**Authors:** Mohamed El Sayed, Kamis Gaballah

**Affiliations:** ^1^Restorative Dentistry Department, College of Dentistry, Ajman University, Ajman, UAE; ^2^Endodontic Department, Faculty of Dentistry, Mansoura University, Mansoura, Egypt; ^3^Department of Oral and Craniofacial Health Sciences, College of Dental Medicine, University of Sharjah, Sharjah, UAE

## Abstract

**Materials and Methods:**

This study was conducted on the mandibular first molars of 54 patients (35 males and 19 females) with signs and symptoms of SIP. To anesthetize the affected molars, all patients received a single carpule of 2% lidocaine with 1 : 100000 epinephrine using a standardized inferior alveolar nerve block (IANB) technique. The cold test was conducted before beginning the endodontic procedures and after gaining lip numbness, and the results were reported as either positive or negative response. The root canal preparation (RCP) was then initiated and the patients' responses were documented (Gold standard test). True pulpal anesthetic failure was described as a pain perception during the access cavity and pulp tissue removal. True pulpal anesthesia was defined as no pain or discomfort during the access cavity and pulp extirpation. The qualitative variables frequencies and percentages of patients with true/false positive and negative responses were determined and then compared using the Chi-square test. The pain perception of male and female patients during the cold test and gold standard was compared using the Fisher exact test. The following diagnostic parameters were calculated using an online statistical calculator: sensitivity, specificity, predictive values, accuracy, and Youden index. In addition, a receiver operating characteristic curve (ROC) was constructed and the area under the curve (AUC) was calculated.

**Results:**

The overall percentage of actual failure of pupal anesthesia was 57%. The sensitivity, specificity, positive predictive value, negative predictive value, accuracy, and Youden index for the cold test were 0.87, 0.91, 0.93, 0.84, 0.89, and 0.78, respectively. There was no statistically significant difference between male and female patients regarding their responses to cold testing and the gold standard test (*P* > 0.05). Besides, the patients' reactions to the cold test were significantly matched with their reactions to the gold standard test (*P* < 0.05). The area under the ROC was mostly 0.9.

**Conclusion:**

The cold test could be a valuable and accurate method for predicting the potential pupal anesthesia before beginning the endodontic treatment of mandibular molars with symptomatic irreversible pulpitis, particularly after obtaining postanesthetic soft tissue numbness.

## 1. Introduction

Local anesthesia is the main method of pain control in dental practice. Difficulty experienced in obtaining adequate pulpal anesthesia with regional blocks is not uncommon, particularly during root canal treatment (RCT) [1]. Several studies found that the effective anesthesia of pulpal tissues was challenging, particularly in mandibular teeth with inflamed pulp [2, 3]. In a previous study, more than 70% of patients who received an inferior alveolar block (IANB) reported inadequate anesthesia for their mandibular molars [4]. Others found a correlation between inflamed pulp tissue and lower pain thresholds in patients, possibly due to increased vascular permeability and accumulation of local inflammatory mediators [5]. Inflammation of pulpal tissues can also increase resistant sodium channels which reduce the efficacy of local anesthesia [6]. Additionally, it has been reported that nerve impulses continue to propagate in acutely inflamed pulps after anesthetic injection [6, 7].

The clinical anesthetic depth evaluation and the prediction of pulpal anesthetic failure before commencing the RCT are challenging, particularly in patients with SIP [7, 8]. Unpredicted pain can induce discomfort and distress during and after the endodontic treatment procedures [9]. Historically, the efficacy of anesthesia with the mandibular nerve block was measured in a sense of “lip and tongue edge numbness” [6]. It was believed that if soft tissue anesthesia was obtained, which was manifested by the absence of mucosal reactivity to a sharp instrument, the dental pulp anesthesia would also be adequate. The conventional current testing of pulpal anesthesia is affected by many factors such as the patient's anxiety, previous experience, and health [8]. Several studies have confirmed that the numbness of the soft tissue is not a guarantee of effective pulpal anesthesia [9, 10]. Walton and Abbott found that 84% of the 268 patients experienced pain during endodontic treatment despite receiving postanesthetic soft tissue signs [3].

Because of the weak correlation between soft tissue anesthesia and true pulpal anesthesia, more comprehensive approaches should be considered for assessing pulpal anesthesia. Using both the electrical pulp test (EPT) and cold test, the efficacy of various local anesthetics and their delivery methods were clinically evaluated [10–12]. Such studies concluded that that deep anesthesia or painless procedures could be predicted by the negative response to the cold test. Few trials tested the efficacy and efficiency of various pulp sensibility tests to ascertain the adequacy of block anesthesia before dental therapy. However, these studies were not consistent with their methods and the subsequent outcomes [13–15].

Sensitivity (SN), specificity (SP), accuracy (AC), positive predictive values (PPV), and negative predictive values (NPV) were used to evaluate the pulpal diagnostic tests as tools for predicting profound pulpal anesthesia [16–20]. All of these parameters must be compared to the gold standard test, which is the single best test that can be used to determine if anesthesia was successful or not. To determine these diagnostic parameters, the number of patients with true positive (TP), false positive (FP), true negative (TN), and false negative (FN) results must be determined [16]. If the test result is yes or no, or positive or negative, sensitivity and specificity are widely used to assess the accuracy of a diagnostic test [16].

Sensitivity (TP/TP + FN) refers to the likelihood that the test will correctly classify the presence of the problem (failure of pulpal anesthesia), while specificity (TN/TN + FP) refers to the likelihood that the test will correctly classify the absence of the problem (success of pulpal anesthesia) [21]. The sensitivity and specificity of a diagnostic test vary from 0 to 1, with 1 indicating the test can correctly identify all the diseased subjects (failure of pulpal anesthesia) as diseased and all the nondiseased subjects as nondiseased (profound pulpal anesthesia). Sensitivity and specificity have an inverse relationship, which means that as sensitivity increases, specificity decreases and vice versa [22].

The percentage of patients with a specific problem (abnormal condition such as disease or failure anesthesia) and who have a positive response to a diagnostic test is known as positive predictive value (PPV). If the test outcome is positive, this measurement equals the likelihood of patients experiencing a specific problem (such as failure anesthesia). The PPV (TP/TP + FP) indicates how many of the test positives are true positives; if this amount is higher (as close to one as possible), the test is performing as well as the gold standard [23]. The negative predictive value (NPV) is the percentage of average patients (with normal conditions such as good anesthesia) who have a negative response to a diagnostic test. The NPV (TN/TN + FN) indicates how many test negatives are true negatives [23, 24]. The accuracy of a test (TP + TN/TP + TN + FP + FN) dictates how well it can detect the presence or absence of a problem [24].

Youden's index (YI) is another diagnostic parameter proposed by Youden to determine the overall benefit of a dichotomous diagnostic tool [25]. Ruopp et al. explained the Youden index as the uppermost possible effectiveness of a diagnostic test [26]. It is a straightforward equation that involves adding a diagnostic test's sensitivity to its specificity, then subtracting 1 from the result. The diagnostic test meets scientific standards for use as a diagnostic tool if the Youden index is greater than 50%.

Constructing the receiver operating characteristic (ROC) curve is considered a valuable method to quantify the accuracy of a medical test by estimating the area under the curve (AUC) [20]. The AUC can be estimated using a variety of parametric or nonparametric distributional assumptions [20]. It also is possible to test the AUC against the null hypothesis of chance performance, or that the AUC in the population is 0.50 [27].

The utilization of the cold test to evaluate the pulpal anesthesia of extremely inflamed pulp before commencing the RCT was not adequately investigated with verification of this test during the access cavity and pulpectomy procedures. This research was carried out to investigate the efficacy of the cold sensibility test, in comparison to the gold standard test, to predict the profound pulpal anesthesia before starting the RCT of lower first molars with symptomatic irreversible pulpitis. The current study's null hypothesis was that there was no significant difference between the findings of the postanesthesia cold test and the gold standard test.

## 2. Materials and Methods

### 2.1. Study Design and Patient Selection

The current cross-sectional clinical investigation was designed according to the STARD guidance for reporting diagnostic accuracy [28] and after getting the ethical board approval of Ajman University, UAE. One hundred-nineteen adult patients (18–40 years old) reported to the University Clinics were invited to take part in the study. The diagnostic and treatment procedures were clarified to patients, and intentionally written consent was given by all participants. All participants were informed that they could exit the study at any time without implications or violation of any prior rights. The inclusion criteria to enroll in the study were as follows: patients must have symptoms of symptomatic irreversible pulpitis in the first mandibular molar due to caries or defective direct restoration [29]. The patients should have a recent history of acute spontaneous pain categorized as moderate (scale 4–6) to severe (scale 7–10) based on the 0–10 Numerical Rating Scale (NRS) [30]. This scale was used to eliminate any bias caused by varying levels of pain durings' selection. The exclusion criteria were as follows: pregnancy (female patients), gross caries rendering the tooth unrestorable, teeth with artificial crowns, advanced periodontitis, radiological evidence of root resorption, teeth with narrow pulp chambers, cracked teeth, patients with a history of significant adverse reaction to local anesthetics including the allergy, uncontrolled diabetes or hypertension, intake of drugs that may interfere with sensation in the orofacial region, severe dental/needle phobia, and inability to give informed consent. Moreover, the study did not include patients with delayed or lack of response to cold testing, the presence of extensive periapical pathologies, or necrotic coronal pulp tissue that occurred while the pulp chamber was being penetrated.

### 2.2. Diagnosis Confirmation

The start time was standardized to be between 9.00 and 11.00 am for all clinical measures. The principal investigator checked the initial diagnosis and sensibility of the pulp using the cold test at the start of each appointment and before giving local anesthesia. Cotton rolls were used to partially isolate the affected and adjacent teeth, and a cotton pellet # 2 saturated with Green Endo-Ice refrigerant (Coltène/Whaledent Inc., OH, USA) was applied for 5 seconds to the mid-third of the buccal surface tooth's crown. Besides, the sensibility of adjacent teeth was also examined. Participants were asked to report their responses in the form of a feeling of pain in their teeth. The patients' responses were recorded based on the 0–10 Numerical Rating Scale [30]. If the appalling reaction to the cold tests was severe and persistent compared to a contralateral molar tooth, then the case was diagnosed as SIP. The diagnosis obtained was verified by the lack of radiological signs of periapical pathosis.

### 2.3. Local Anesthetic Procedure

A total of 79 patients who met the inclusion and exclusion criteria were initially recruited in the study and received an inferior IANB using 1.8 ml of 2% lidocaine with 1 : 100,000 adrenaline (Dentsply Pharmaceutical, USA). The injection was done over 20 seconds usinga 27-gauge long needle. All patients were initially asked after 5 minutes about the possibility of lip numbness sensation. Lip numbness was confirmed by gently pricking the lip surface two times at points close to the midline to the mouth commissure on the same tested side. The patients who showed lip numbness and a negative response to these stimuli within 15 minutes after anesthetic injection were assessed as IANB success and were finally assigned for this study (*N* = 54). Patients who did not show lip numbness or positive response to the picking stimuli within 15 minutes were excluded from the study and treated by another specialist (*N* = 25).

### 2.4. Samples Size Determination

The sample size was determined based on the statistical formula stated by Chavarría-Bolaños et al. [4]. The following formula is described as follows:

(1) $$\begin{array}{c} n=\frac{{\left[Z_{\alpha }\;/2\sqrt{2P\left(1-P\right)}+\;Z_{\beta }\sqrt{P1\left(1-P1\right)+P2\left(1-P2\right)}\right]}^{2}}{{\left(P1-P2\right)}^{2}}, \end{array}$$

where *Z*_*α*_ is standard and equal to 1.96 and *Z*_*β*_ is standard and equal to 0.84 when the sample size calculation was performed with a type I error of 0.05 (significance of 95%) and statistical power of 80%. *P* is the average of sensitivity percentage values of the cold test (0.84) and gold standard (1.0) as reported in a previous study [31]. *P*1 is the percentage value of sensitivity of the gold standard in the previous study [31]. *P*2 is the percentage value of sensitivity of the cold test in the same previous study [31].

After applying exclusion criteria and based on the previous formula, the required sample size was 44 patients however, fifty-seven patients were included in the current study. This number of patients was deemed sufficient to demonstrate any differences that could be attributed to the diagnostic tests used.

### 2.5. The Postanesthetic Cold Test

After 15 min, the cold sensibility test was performed to identify the presence of pulpal anesthetic failure, and the results were compared to the gold standard test. The gold standard benchmark for anesthetic failure was identified as any painful sensation or discomfort during the access cavity preparation and the pulpal tissue manipulation. The level of pulp removal was standardized until a full pulpectomy was accomplished.

The target tooth was fully isolated using a rubber dam and then subjected to the cold test as stated previously after the soft tissue anesthesia was verified. After a one-minute interval, the test was repeated two more times. Regardless of the level of pain, the painful response or any discomfort of patients at any level of pulpectomy procedures was registered as a positive response.

### 2.6. Patients' Responses during Access Cavity and Pulp Extirpation

Under rubber dam isolation and air-water coolant, the access cavities were done using Endo Access Kit (Dentsply Tulsa Dental Specialties; Tulsa, Oklahoma). The enamel and dentin were penetrated using a new high-speed diamond round bur #4. Once the pulp chamber was approached, a complete deroofing was performed using Endo Z bur and the pulpectomy was carried out. Whatever its severity, any painful reaction or discomfort during the access cavity and pulpectomy procedures was reported as a positive or negative response. The main criterion for the real pulpal anesthetic failure was pain or discomfort perception during the access cavity and pulpal tissue removal. The pain or discomfort sensation was reported when the patient raised their hand. To alleviate their symptoms, all assigned patients had a first appointment that involved the removal of all pulpal tissue from the pulp chamber and root canals, followed by a second appointment for root canal preparation and obturation. If the patients had a painful reaction during the access cavity or pulp extirpation, an additional carpule of lidocaine was administered as a second IANB or intra-pulpal injection, and the endodontic procedure was resumed painlessly. All endodontic procedures were conducted by the principal investigator, who also reported the patients' responses.

### 2.7. Statistical Analysis

Patients' responses to the postanesthetic cold test and gold standard test were measured, and the findings were statistically evaluated using the Pearson Chi-square test. The pain experiences of male and female patients during the postanesthetic cold test and the gold standard test were compared using a two-tailed Fisher exact test. The outcomes of these results were considered significant at *P* < 0.05.

The numbers of patients who responded to the postanesthetic cold test with true positive (TP), false positive (FP), true negative (TN), and false negative (FN) responses were calculated and compared to the results of the gold standard test.  Table 1 summarizes the meanings of these parameters. Using MedCalc's free online “diagnostic test statistical calculator,” sensitivity (SN), specificity (SP), positive predictive value (PPV), negative predictive value (NPV), and accuracy (AC) with confidence intervals (95% CI) were calculated for the cold test [32]. Youden index was determined for the overall diagnostic precision of the postanesthetic cold test using the following equation: specificity + sensitivity − 1 [25].

Finally, for the cold test, a receiver operating characteristic (ROC) curve analysis was used to measure overall predictive power and quantify the region under the curve (AUC) [33, 34]. All statistical analysis (Chi-square, Fisher exact test, and ROC curve) was performed using version 20 of the SPSS software program (IBM, Armonk, NY, USA).

## 3. Results

**Figure 1:**
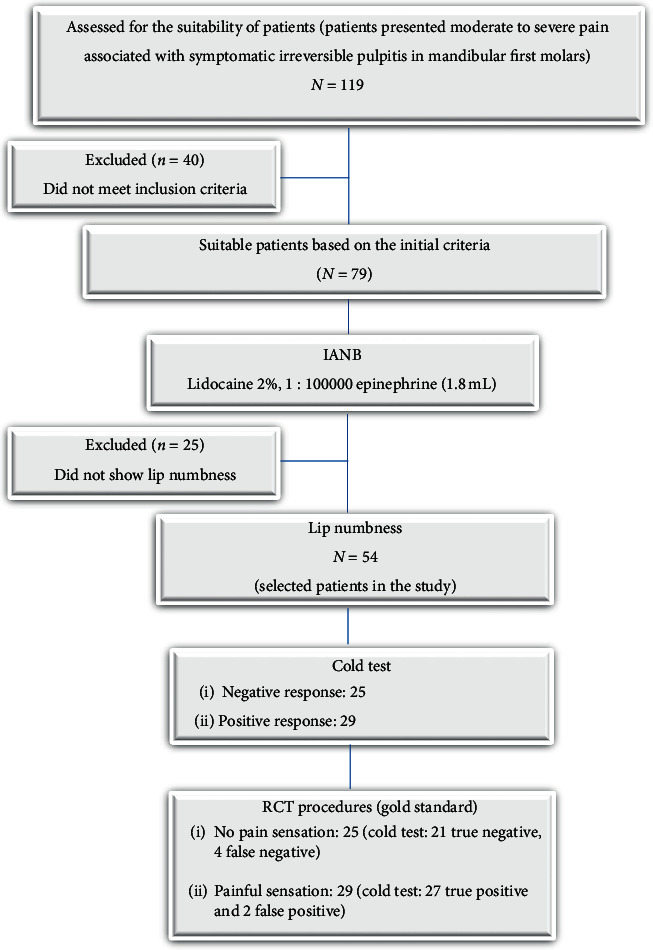
A flowchart of the clinical study.

The final research sample consisted of 54 adult participants, 35 of whom were males (65%) and 19 of whom were females (35%). The participants' ages ranged from 18 to 40, with an average of 32.3±5.5 years.  Figure 1 illustrates a graphic representation of the clinical trial and its outcomes. There were no requests for patients to withdraw from the study, and no adverse effects were observed during the current study. All selected patients showed successful IANB anesthesia based on their profound soft tissue signs within 15 minutes after anesthetic injections. The postanesthetic cold test and the gold standard test, on the other hand, showed 53.7% and 57.4% IANB failure, respectively (Table 2). In terms of the results of the postanesthetic cold test and the gold standard test, there was no statistically significant difference (*P* > 0.05) between male and female patients (Table 2). The Chi-square test (Table 3) showed a strong association between the results of the postanesthetic cold test and the gold standard (*P* < 0.05). Based on these results, the current study's null hypothesis was accepted.  Table 4 shows the true/false positive and true/false-negative outcomes of the cold test and gold standard. In contrast to the gold standard, the cold test revealed two patients with false-positive responses and four patients with false-negative responses. As a result, the total number of false results obtained with the postanesthetic cold test was 11%.

**Figure 2:**
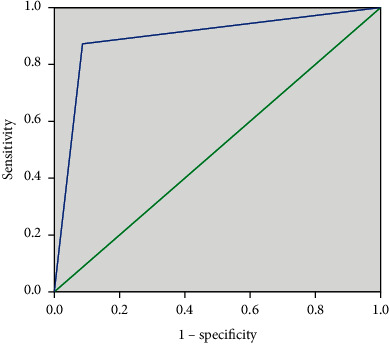
Receiver operator characteristic curve (ROC) for the cold test.

Table 5 shows the diagnostic parameters' results of the postanesthetic cold test. The sensitivity and specificity of the cold test were 0.87 and 0.91, respectively. A positive response to the postanesthetic cold test predicted 87% of teeth with failed pulpal anesthesia (as manifested by pain during endodontic procedures), while a negative response predicted 91% of teeth with effective pulpal anesthesia. The PPV and NPV, respectively, were 0.93 and 0.84. Thus, a positive response to the cold test suggested a pulpal anesthetic failure with a 93% likelihood, and a negative response to the postanesthetic cold test indicated profound pulpal anesthesia with an 84% probability. The cold test had an accuracy of 89% and YI value was 0.78. The ROC curve is graphically illustrated in Figure 2 and AUC results are presented in Table 6. The area under the curve (AUC) was 0.925 with *P* < 0.05.

## 4. Discussion

IANB anesthesia currently remains the preferred approach adopted by dental practitioners to achieve a pain-free dental operation on the mandibular molar teeth. Previous clinical studies indicated that anesthetic failure in mandibular molars occurs frequently in patients with SIP [35, 36]. Failure of IANB to achieve a profound working pulpal anesthesia resulted in several studies focused on assessing the efficacy of anesthesia before commencing the operative dental procedures.

The mechanical response to proprioceptive nerve stimulation can be evaluated by the presence of tissue numbness after performing anesthesia. Therefore, the operator could only consider the IANB as successful after blocking the lip and cheek proprioception sensation. The numbness of the soft tissue, therefore, does not automatically reveal pulpal anesthesia [36, 37]. Several research reports suggested that lip numbness is a poor indicator of IANB success and should not be clinically used [38–41]. Consequently, some researchers suggested the use of thermal or electrical pulp stimulation as impartial measures for pulpal anesthesia evaluation [38–42]. The cold sensibility test is considered a regular diagnostic aid for the detection of pulp sensibility [43]. This test is based on the low-temperature transfer from a cotton pellet saturated with a refrigerant to the dental pulp via the hard-dental tissues [43, 44]. Also, the incompletely anesthetized pulp may elicit a negative response to the cold test at the level of peripheral nerve endings (A-*δ* fibers) [45]. In contrast, the cold test does not activate the deeper pulp areas, which are rich in C-fiber nerve endings, and a false negative result is obtained. Consequently, the actual sensibility to the pulp cannot be reported accurately [45]. The same scenario could explain pain perception during the endodontic access cavity preparation, where pain-free penetration of the dentin can conceal a failed IANB due to inadequate depth of anesthesia. The presence of severe pulpal inflammation, particularly in the deep areas, may justify this clinical condition based on the acidic pH, overexpression of abnormal sodium channels, an increase of inflammatory pain mediators, and abnormal vasodilation [35, 38].

Most clinicians rely on targeted soft tissue numbness and pain-free penetration with a sharp explorer into the tissue to check the performance of block anesthesia. Nonetheless, based on previous researches, the accuracy of this technique was not reliable and so it is important to look for an objective test to assess block anesthesia [36–41]. The ideal method for determining pulp status should be nondestructive, painless, standardized, reproducible, reliable, affordable, simple to complete, and objective [46]. Some researchers suggested using thermal or electrical stimulation as more objective measures to assess pulpal anesthesia [38, 42, 47, 48]. However, the ability of the cold test to assess anesthetic performance has not been adequately investigated. Therefore, the present clinical study was designed to investigate the consistency of the cold sensibility test to anticipate the failure and success of IANB during endodontic treatment of mandibular first molars with SIP.

Green Endo-Ice has been chosen to test pulpal anesthesia because it has many advantages, such as being easy to use, inexpensive, quick to conduct and well-endured by most patients. Green Endo-Ice is composed of 1,1,1,2-tetrafluoroethene (TFE) and has a lower temperature (−26.2°C) than regular ice or ethyl chloride. Also, the cold sensibility test is considered highly effective in assessing pulpal vitality [49]. Some investigators assessed the dichlorodifluoromethane (DDM) refrigerant as a pulp sensibility tester. However, due to its potential environmental hazard, this product is no longer commercially available and has been replaced by TFE [49]. Another refrigerant named Endo-Frost or Endo-Ice F (−50°C) (Coltène/Whaledent) was introduced in the market and usually contains 30–50% propane, 30–50% butane, and 10–20% isobutane. Both Endo-Ice F and Green Endo-Ice have similar reduction effect on the temperature inside the pulp chamber [49].

The pain assessment during the access cavity and removal of pulp tissues will provide the clearest evidence of the presence or absence of pulpal anesthesia [37]. As a result, this criterion was used as a gold standard in this study to determine pulpal anesthesia and compare the results of postanesthetic cold tests. Pain perception in response to pulpal anesthetic failure during access cavity and pulpal tissue removal differs from person to person, varying from light sensation to severe discomfort. To reduce subjectivity in the current study, regardless of responsiveness, the duration and intensity of the response were not assessed, and only positive or negative reactions were addressed during the postanesthetic cold test and gold standard test. The primary goal of local anesthesia is to perform painless endodontic procedures to reduce the patient's stress and increase his confidence. Furthermore, deep pulpal anesthesia during the endodontic procedures decreases the postoperative pain and may reduce the need for analgesic drugs [4, 50]. All patients who were chosen for the present study submitted their consent and were told that the tests that were used could cause pain or discomfort. Failure of anesthesia can result in unpleasant pain, which is considered a normal reaction [4]. Several investigators recorded a 30–90% probability of anesthetic failure when administering a single IANB in patients with the acutely inflamed pulp [1, 51, 52]. In the case of failed pulpal anesthesia, most of those studies reported lip numbness.

The results of the current study are consistent with previous reports that showed great difficulty in accomplishing the profound pulpal anesthesia for mandibular molar with irreversible pulpitis [44, 53, 54]. The failure rate of IANB in the present study was 57% based on the gold-standard test. The pulpal anesthetic failure may be due to the presence of pulpal inflammation, abnormal neural anatomy, and patients' anxiety [1, 6, 55, 56]. The clinical significance of this finding is determined by the clinician's ability to detect IANB failure before gaining access to the dental pulp and causing undue discomfort to the patient.

The statistical analysis showed no significant difference between male and female patients about pain perceptions during the endodontic procedures. This result substantiated the published data on 83 patients, which indicated that the sex of patients did not affect pain perception during root canal treatment [39].

The findings of the current study indicated that IANBs had successfully anesthetized the pulp tissues when patients had a negative reaction to the postanesthetic cold test. The cold test's sensitivity for identifying pulpal anesthetic failure was high (87%). The cold test's specificity for identifying pulpal anesthetic success was also high (91%). The percentage of false-negative responses (7.4%) in the current findings, however, is lower than those of some studies [14, 24] but higher than other previous findings [44]. This disparity may result from the use of various pulp sensitivity tests to evaluate pulpal anesthesia. Some authors [6, 14, 24] used EPT which was found to be less accurate and responsive than the cold test. Other scientists [4, 44], on the other hand, used DDM or Endo-Frost, providing −50°C compared to −26°C from the TFE test used in the present study.

The current results revealed that both PPV and NPV values were the same as 1.00 for the gold standard test, and 0.93 and 0.84 for the postanesthetic cold test, respectively. Clinically, the PPV indicates the IANB failure is likely every time the results of the test are positive. According to the current study's PPV (0.93), 93% of patients who have a positive response to cold test are likely to have true anesthetic failure. The NPV was defined as the percentage of subjects with a negative response to the cold test who truly had a successful IANB (lack of sensation during endodontic procedures). According to the NPV in the present study (0.84), anesthetic success can be expected in 84% of patients with a negative response to the cold test. Despite the high NPV value in the present study, anesthetic failure occurred with a negative reaction to the cold test in a small proportion of patients (16%). The present findings showed the cold test was capable of detecting IANB failure (SN = 0.87 and total performance (accuracy) = 0.89). When the ROC curve and AUC score (0.892) of the cold test were analyzed, responsiveness for this test showed high accuracy as the AUC value is closely near 0.9 [24, 27].

In the present study, Youden index and receiver operating characteristic curve (ROC) were used assessed to evaluate the general performance of the postanesthetic cold test. Youden index is a single and simple statistic that summarizes the performance of a dichotomous diagnostic test and it is often used in conjunction with receiver operating characteristic (ROC) analysis [57]. This test combines sensitivity and specificity into a single measure (sensitivity + specificity − 1) and has a value between 0 and 1. In a perfect test, the Youden index equals one [58–60]. A diagnostic test is considered successful if its Youden index is greater than 0.5. [59]. The measured value of the Youden index in the present study was 0.78, indicating that the postanesthetic cold test has an outstanding overall efficiency in detecting pulpal anesthesia.

Previous randomized clinical trials have shown that patients with SIP mandibular molars and negative response to cold testing may still reveal variable levels of pain during endodontic treatment [38, 47]. Many studies have evaluated the accuracy of pulp sensibility tests by measuring their sensitivity, specificity, and predictive values [13, 15, 47]. The results of these studies revealed that the cold test has high accuracy in the determination of dental pulp sensibility. Hsiao-Wu et al. [39] calculated the sensitivity, specificity, and predictive values of the postanesthetic cold test as indicators of successful pulp anesthesia, with values of 0.58, 0.67, 0.88, and 0.38, respectively. We have achieved higher values for sensitivity, specificity, and negative predictive values. However, the positive predictive values of the current study and Hsiao-Wu et al. [39] were almost similar. Furthermore, the current findings contradict the findings of a similar study conducted by Chavarría-Bolaños et al. [4]. These inconsistencies may be attributed to differences in the study design and selection of the gold standard test. Chavarría-Bolaños et al. compared the cold test to lip numbness and cavity preparation procedures, using the pulpal manipulation only as a gold standard [4]. Also, the administration of local anesthesia in that study was done by postgraduate endodontic residence [4]. The anesthetic solution used in their study was mepivacaine 2% with 1 : 100000 epinephrine, which has a delayed onset, on mandibular first and second molars [4]. Furthermore, those authors [4] used the Endo-Frost as a diagnostic test which has too much lower temperature (−50°C) than the Green Endo-Ice (26.2°C). In the current study, the gold standard test was any pain perceptions during the access cavity and pup tissue manipulation. When the patient experienced pain during dentin penetration, the result was recorded as a positive response, indicating true anesthetic failure and the need to penetrate further. However, in this case, an additional carpule of anesthetic solution was administered as a supplemental injection, and the endodontic procedures were completed without further testing. The anesthetic solution used in this study was 2% lidocaine with 1 : 100,000 adrenaline, which has a rapid onset of action, and the injection was performed by a consultant surgeon. Furthermore, because lip numbness was not considered a test variable in the current study, any patient who did not have profound lip numbness within 15 minutes was excluded.

Lip numbness and pulpal anesthesia are signs of IANB success. Lip numbness means that the anesthesia inhibits pressure sensitization, but this is not always the case for pulpal anesthesia [36]. The cold test, which relies on temperature transmission from a cotton pellet to the dental pulp through enamel and dentin, is a well-known technique for detecting pulp sensibility [16, 43]. Furthermore, although the thermal response of partially anesthetized pulp can elicit a negative response in peripheral nerve endings (A-fibers), the deeper pulp areas, which are rich in C-fiber nerve endings, may not be stimulated by cold and may go unnoticed [61]. This can explain the presence of false-negative responses in the current study. A situation similar to this could occur during dentin penetration, where the painless removal of peripheral dental structures could mask a failed IANB due to an insufficient anesthetic effect. Based on a lower local pH, TTX-r sodium channel overexpression increased prostaglandins E2 levels, and increased vasodilation, preoperative inflammation of the dental pulp, particularly deep pulpal tissue, may explain this behavior [13, 35].

The drawback of this study was the lack of a psychological assessment during the recruitment interview that could be considered in future work. Despite efforts to select a uniform cohort of participants, individual perceptions of pain and treatment techniques may also affect the response to local anesthesia. The time to finish the access cavity and to remove all pulp tissue from the pulp chamber and root canals was not standardized and this may affect the result of the gold standard test. The emotional response to pain may be correlated with the pessimistic perception of pain-associated fears [56] and may influence the outcome of the investigation [24].

Finally, the dentists must verify the depth of the IANB beforehand to anticipate the potential of anesthetic failure during the treatment of mandibular molars with SIP that may reflect on the psychological behavior of patients during and after treatment.

## 5. Conclusions

Failure to obtain profound pulp anesthesia following IANB of mandibular molars with SIP is significant in 57% of patients even with profound soft tissue anesthesia. The postanesthetic cold dental test is sensitive (87%) and specific (91%) enough to predict the depth of pulp anesthesia following IANB in patients with symptomatic irreversible pulpitis of mandibular first molars. The cold test, along with soft tissue anesthetic signs, should be considered as a diagnostic test to predict IANB 's success before starting root canal therapy of mandibular first molars with irreversible pulpitis to minimize pain and anxiety during clinical procedures.

## Figures and Tables

**Table 1:** Definitions of true/false positive and true/false negative responses to the cold test.

Terminology	Definitions
True positive response (TP)	A positive response to postanesthetic cold test and painful endodontic procedures
True negative response (TN)	A negative response to postanesthetic cold test and pain-free endodontic procedures
False positive response (FP)	A positive response to postanesthetic cold test and pain-free endodontic procedures
False negative response (FN)	A negative response to postanesthetic cold test and painful endodontic procedures

**Table 2:** Comparison between pain perception of male and female patients to postanesthetic cold test and gold standard test, and the overall percentage of pulpal anesthetic failure and success.

Gender (total number = 54)	Patients' responses-*N* (%)			
	Cold test		The gold standard test (procedures test)	
	Pain (predicted failure)	No pain (predicted success)	Pain (true failure)	No pain (true success)
Males (*N* = 35/65%)	17 (49%)	18 (51%)	19 (54%)	16 (46%)
Females (*N* = 19/35%)	12 (63%)	7 (27%)	12 (63%)	7 (27%)
Total-*N* (%)	29 (53.7%)	25 (46.3%)	31 (57.4%)	23 (42.6%)
*P* value^*∗*^	0.3952		0.5766	

^*∗*^Two-tailed Fisher exact test (difference *P* < 0.05).

**Table 3:** Statistical analysis of postanesthetic cold test results versus gold standard test results.

Test results			Gold standard		Total
			Positive	Negative	
Cold test	Positive	Count	29	2	31
		Expected count	18.9	12.1	31.0
	Negative	Count	4	19	23
		Expected count	14.1	8.9	12.0
Total		Count	33	21	54
		Expected count	33.0	21.0	54.0
Pearson Chi-square test		*χ* ^2^	32.223		
		df	1		
		Sig. (*P* value)	0.000		

**Table 4:** Number and percentages of patients with true/false positive and negative responses to the postanesthetic cold test versus the gold standard test.

Test	Results	True results based on the gold standard test *N* (%)^†^		Total-*N* (%)
		Overall true positive *(no pulpal anesthesia)*	Overall true negative *(pulpal anesthesia)*	
Cold test	Positive *(painful response)*	True positive (TP) 27 (93%)	False positive (FP) 2 (7%)	29 (53.7%)^*∗*^
	Negative response *(painless response)*	False negative (FN) 4 (16%)	True negative (TN) 21 (84%)	25 (46.3%)^*∗∗*^
Total-*N* (%)		(True failure anesthesia) 31 (57.4%)	(True successful anesthesia) 23 (42.6%)	54 (100%)

^†^ *N* (%): number and frequency of patients. ^*∗*^Failure pulpal anesthesia as indicated by the cold test. ^*∗∗*^Successful pulpal anesthesia as indicated by the cold test.

**Table 5:** Results of sensitivity, specificity, positive predictive value, negative predictive value, accuracy, and Youden index for the cold test with confidence intervals 95% (CI 95%) and at 57% prevalence of IANB failure.

	Estimated value	95% confidence interval	
		Lower limit	Upper limit
Prevalence	0.57 (57%)	0.433	0.705
Sensitivity	0.87 (87%)	0.702	0.964
Specificity	0.91 (91%)	0.719	0.989

*For any particular test result, the probability that it will be:*			
Positive	0.54 (54%)	0.397	0.672
Negative	0.46 (46%)	0.328	0.603

*For any particular positive test result, the probability that it is:*			
True positive (positive predictive value-PPV)	0.93 (93%)	0.781	0.981
False positive	0.07 (7%)	0.012	0.242

*For any particular negative test result, the probability that it is:*			
True negative (negative predictive value-NPV)	0.84 (84%)	0.676	0.930
False negative	0.16 (16%)	0.052	0.369
Accuracy	0.89 (89%)	0.774	0.958
Youden index	0.78 (78%)	0.421	0.953

**Table 6:** Area under the curve (AUC) from receiver operator characteristic curve analysis.

Area	Std. error^a^	Asymptotic sig.^b^	Asymptotic 95% confidence interval	
			Lower bound	Upper bound
0.892	0.049	0.000	0.796	0.988

^a^Under the nonparametric assumption. ^b^Null hypothesis: true area = 0.5.

## Data Availability

The data (measurements of inhibition zones) used to support the findings of this study will be available from Dr. Mohamed Elsayed at m.elsayed@ajman.ac.ae for the researchers who meet the criteria to access these data. The data can be requested after the publication of this article. However, requests for the data (6/12 months after the publication of this article) will be considered by the corresponding authors.
